# When the Esophagus Disrupts the Rhythm: New-Onset Atrial Fibrillation After Stent Placement

**DOI:** 10.7759/cureus.87152

**Published:** 2025-07-02

**Authors:** Roger Lin, Milan Regmi, Humza Syed, Ahsan Masood

**Affiliations:** 1 Internal Medicine, Southeast Health Medical Center, Dothan, USA

**Keywords:** atrial fib, esophageal cancer, esophageal mass, esophageal stent, esophageal stricture

## Abstract

Atrial fibrillation (AF) is a recognized complication following esophagectomy and radiotherapy for esophageal cancer but is rarely reported after esophageal stent placement. The mechanisms are thought to include direct mechanical irritation, vagal stimulation, and local inflammation affecting the posterior left atrium. However, the incidence of AF following esophageal stenting remains undocumented. We report the case of an 88-year-old woman with an ulcerated esophageal mass causing dysphagia and weight loss, who developed new-onset AF with rapid ventricular response following esophageal stent placement. The patient had no prior history of arrhythmia and no reversible metabolic or structural cardiac triggers. Telemetry confirmed AF shortly after the procedure. Rate control was achieved with amiodarone. Given recent gastrointestinal bleeding, anticoagulation was deferred at discharge. Her course was further complicated by a lack of follow-up and readmission four months later with progressive decline. This case highlights a rare but important complication of esophageal stenting. The close anatomical proximity of the esophagus to the left atrium may predispose to arrhythmias via mechanical compression and autonomic or inflammatory pathways. While individual case reports have proposed this link, no studies or registries have quantified its incidence. Our case is unique due to the patient’s advanced age, ulcerated mass, and rapid clinical deterioration post-stent. New-onset AF following esophageal stent placement may be underrecognized. Awareness of this potential complication is essential, particularly in patients with limited options for anticoagulation. Future studies should investigate the incidence of AF post-stenting and assess whether targeted monitoring or prophylactic measures are warranted in high-risk populations.

## Introduction

Esophageal cancer is a relatively rare but aggressive malignancy with a significant global burden. In 2020, the worldwide age-standardized incidence rate of esophageal cancer was approximately 6.3 per 100,000 population, with notable geographic variability [1]. Two predominant histologic subtypes-esophageal squamous cell carcinoma and adenocarcinoma-differ in epidemiology and risk factors, but both may exert mass effects on nearby thoracic structures.

Cardiac involvement in esophageal cancer, though uncommon, can have important clinical implications. Tumor invasion or extrinsic compression of the heart, particularly the left atrium, may trigger electrical instability, predisposing patients to atrial arrhythmias such as atrial fibrillation (AF) [2,3]. In a case report by Bayraktar et al., AF was the initial presentation of esophageal cancer in a patient without structural heart disease or conventional AF risk factors, highlighting the arrhythmogenic potential of direct tumor interaction with the myocardium [2]. Additional reports have documented the development of AF following esophageal stent placement, radiotherapy, or esophagectomy, suggesting multiple mechanisms by which esophageal pathology may precipitate arrhythmias [4,5]. Esophageal masses or stents may trigger AF through direct mechanical irritation of the posterior left atrium, enhanced vagal stimulation, and local inflammation-all of which can disrupt atrial electrophysiology. These mechanisms often act synergistically, increasing susceptibility to arrhythmogenesis in affected individuals.

The incidence of AF following esophageal stent placement remains largely undocumented, with no available registries or published studies systematically reporting this occurrence, highlighting the novelty and rarity of such an association. We present a case of a patient with esophageal cancer who developed new-onset AF after esophageal stent placement, in the absence of other known cardiovascular triggers.

## Case presentation

**Figure 1:**
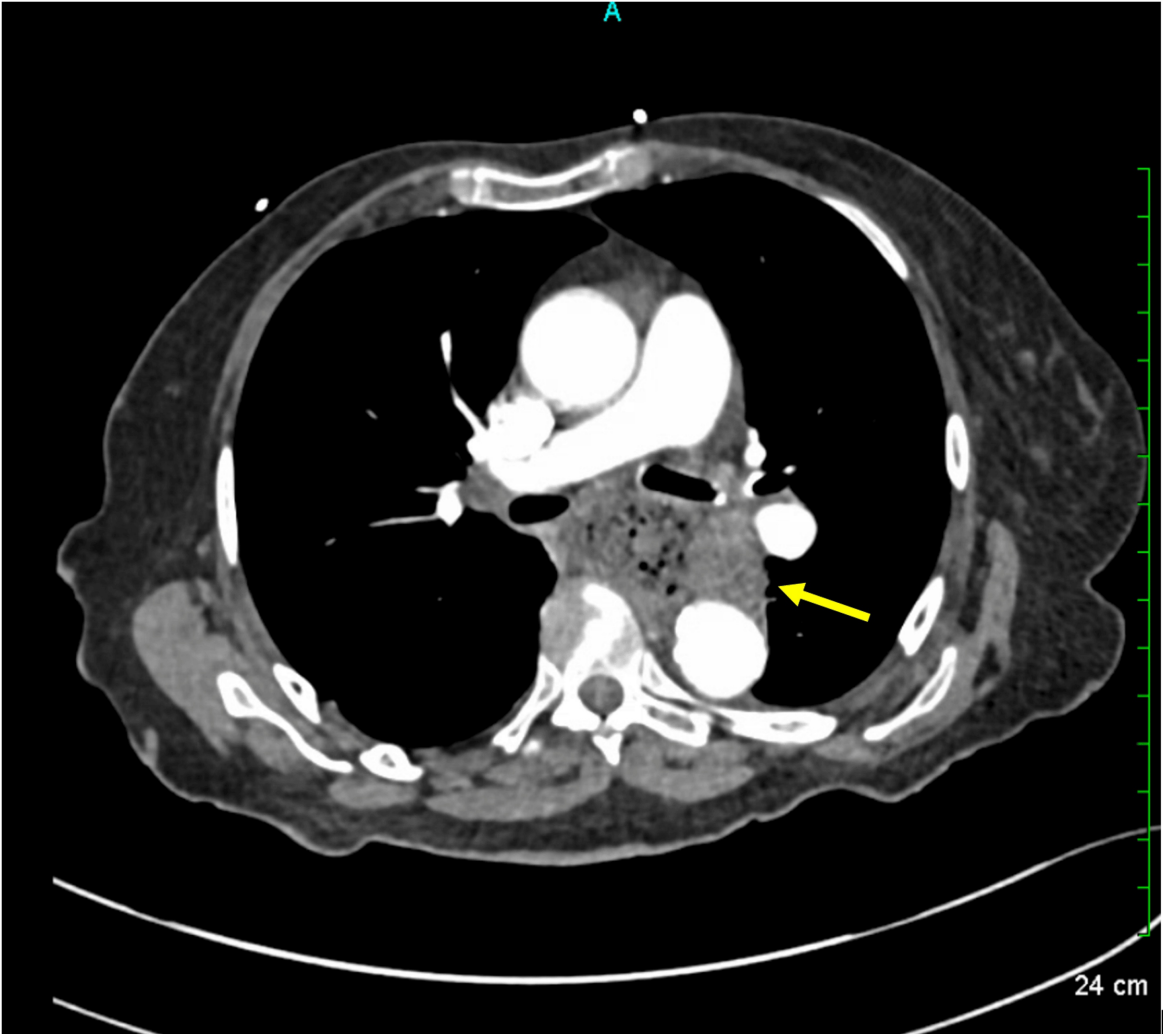
CT of the Chest With IV Contrast Demonstrating an Esophageal Mass Compressing the Trachea and Abutting the Left Atrium Yellow arrow: mass-like thickening involving the distal thoracic esophagus and gastroesophageal junction IV: intravenous

An 88-year-old female patient with a history of well-controlled hypothyroidism, hypertension, and hyperlipidemia presented with progressive dysphagia to liquids and solids, intermittent hematemesis over several months, and an associated 10-pound weight loss. Upon admission, initial laboratory evaluation was notable for troponin of 200 ng/L (reference < 16), total bilirubin of 2.0 mg/dL with indirect bilirubin of 1.73 mg/dL, and lipase of 10 U/L. Complete blood count, basic metabolic panel, and hepatic function panel were otherwise unremarkable. CT imaging of the chest, abdomen, and pelvis with intravenous (IV) contrast revealed mass-like thickening of the distal thoracic esophagus at the gastroesophageal junction, with a large amount of retained content in the proximal esophagus (Figure 1).

**Figure 2:**
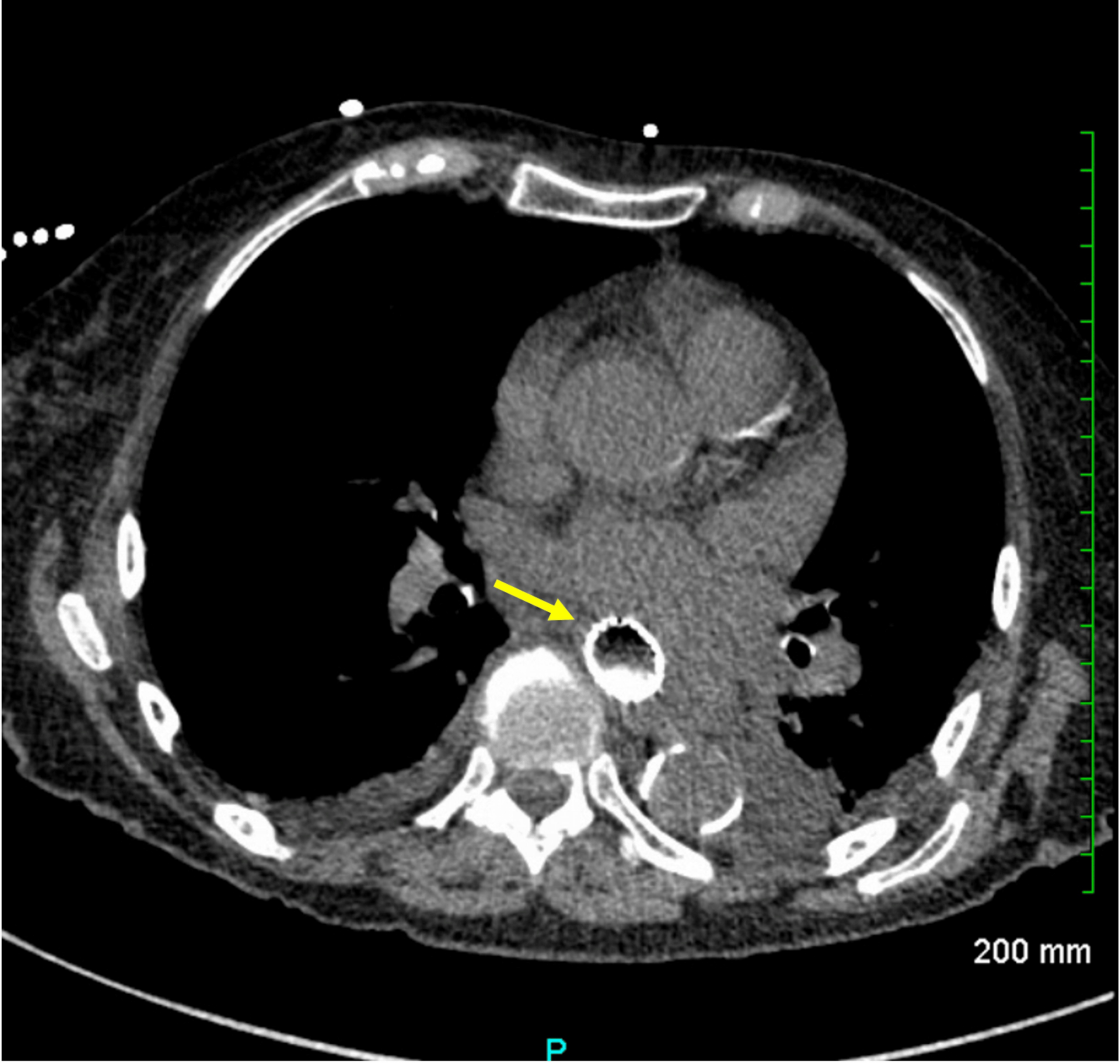
CT of the Chest Without IV Contrast Demonstrating Esophageal Stent In Situ Following Stent Placement Yellow arrow: an esophageal stent is present. This extends from the level just above the carina through the gastric esophageal junction IV: intravenous

**Figure 3:**
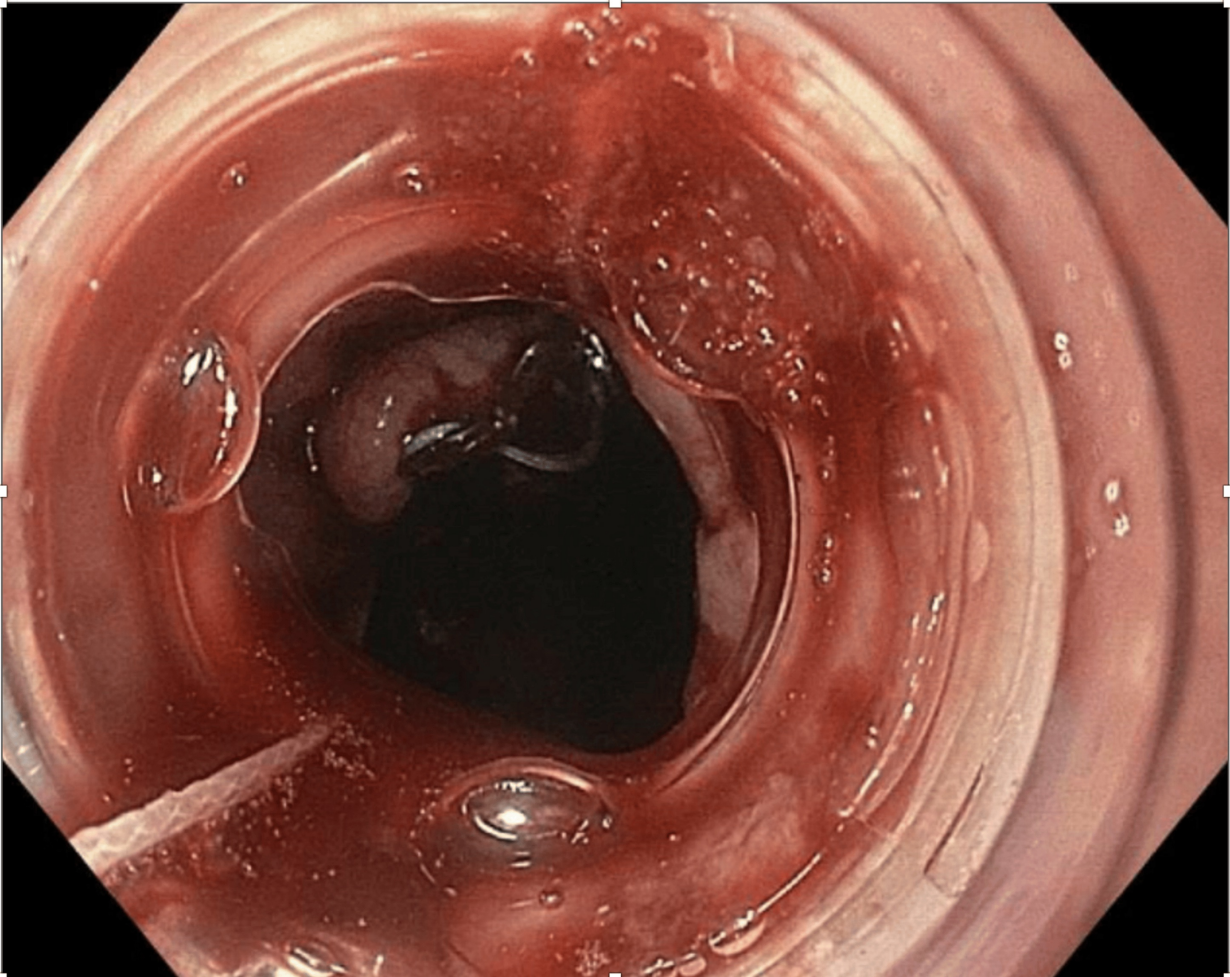
Endoscopic Image of the Esophagus Status Post Esophageal Stent Placement

Esophagogastroduodenoscopy (EGD) was done on the fourth day of hospitalization and identified an ulcerated mass and a malignant-appearing intrinsic stricture in the middle third of the esophagus. Due to severe food and fluid retention from the stricture, esophageal stenting was performed to facilitate drainage and allow enteral feeding (Figures 2, 3).

**Figure 4:**
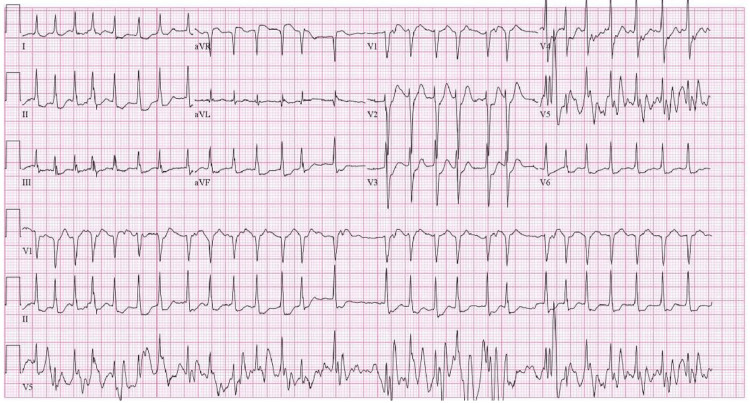
Twelve-Lead ECG Demonstrating Atrial Fibrillation With Rapid Ventricular Response (Ventricular Rate of 165 BPM)

Biopsy revealed necrotic, acutely inflamed, hemorrhagic cellular debris, though suspicion for malignancy remained high. The patient experienced another episode of hematemesis, which resolved with supportive care. On the first day following stent placement, she developed palpitations and dyspnea. Telemetry showed tachycardia, and ECG confirmed AF with rapid ventricular response (Figure 4).

Initial rate control was attempted with a diltiazem drip, but it was discontinued due to hypotension. She was then treated with IV amiodarone followed by a 24-hour loading dose, resulting in conversion to sinus rhythm. Therapeutic Lovenox was held in light of an episode of small volume hematemesis the patient experienced after stent placement, which was controlled later on. Transthoracic echocardiography showed biatrial enlargement, mild to moderate mitral regurgitation, grade 1 diastolic dysfunction, and no severe structural abnormalities. Anticoagulation has had been previously discussed with Gastroenterology. The patient was not a suitable candidate for anticoagulation at the time of discharge due to recent esophageal stent placement and associated bleeding risk. Anticoagulation was deferred, with reconsideration pending Gastroenterology clearance at the outpatient setting. After a 10-day hospitalization, the patient was discharged with amiodarone 200 mg twice daily for 28 days with plans to taper to 200 mg daily and outpatient follow-up with Cardiology and Gastroenterology. The patient did not follow up with Gastroenterology to discuss further management of the esophageal mass. She visited the emergency room four months after being discharged with abdominal pain, nausea, vomiting, and progressive weight loss. However, the patient left against medical advice prior to further workup.

## Discussion

A nationwide population-based study identified esophageal cancer as having the highest association with incident AF among solid cancers [6]. AF has been linked with esophageal cancer following radiotherapy, chemotherapy, and esophagectomy, but AF occurring after esophageal stenting is rare. Mirminachi et al. recently reported a case of AF following esophageal stenting for intramucosal adenocarcinoma [4]. Similarly, our patient developed new-onset AF with rapid ventricular response after esophageal stenting in the setting of an esophageal mass. The patient had baseline risk factors to predispose AF. She has reassuring initial labs, including normal thyroid-stimulating hormone (TSH) at 3.02 U/mL (reference 0.34-4.50), potassium 4.0 mEq/L (reference 3.5-5.1), magnesium 2.0 mEq/L (reference 1.9-2.7), and stable, modestly elevated serial troponins (200, 232, and 211 ng/L; reference < 16), which are more consistent with demand-related myocardial injury rather than primary cardiac pathology. Precluding other identifiable triggers, this highlights that an esophageal mass or stent may precipitate AF by compressing the left atrium or pulmonary vein, inducing ectopic beats in susceptible individuals. This pathophysiology is supported by Mazzella et al., who described reversible AF and atrial tachycardia due to extrinsic left atrial compression, which resolved after esophageal stent removal [5]. Besides the aforementioned mechanism, esophageal masses or stents may trigger AF through several other interconnected physiological mechanisms. The close anatomical proximity of the esophagus to vagal nerve fibers allows esophageal lesions or stents to enhance vagal stimulation, shortening atrial refractory periods and promoting reentrant electrical circuits characteristic of AF. Furthermore, local inflammatory responses elicited by esophageal foreign bodies or tumors can release cytokines and other mediators, altering atrial tissue properties and further increasing susceptibility to arrhythmia. These mechanisms typically act synergistically, collectively heightening the risk of AF in affected individuals.

Research has shown that AF can serve as a surrogate marker for surgical morbidity and mortality following esophagectomy for esophageal or gastric cardia cancer. In one study, mortality in the AF group was nearly four times higher than in controls (23% vs. 6.3%, p < 0.001) [7]. Further research is needed to assess whether AF may similarly predict outcomes following esophageal stenting in patients with esophageal mass or cancer.

Early complications of esophageal stenting include migration, malposition, perforation, and bleeding. AF is a lesser-known but potentially serious complication. Our case reinforces prior reports that vigilance is required for AF after stenting, especially considering the hypercoagulable state often present in cancer patients. The presence of an ulcerating mass in this case posed a relative contraindication to anticoagulation, further complicating AF management.

This case is unique in highlighting new-onset AF in a very elderly patient with an ulcerated esophageal mass and rapid clinical deterioration following stent placement-an association rarely described in the literature. The temporal relationship between stent placement and AF onset raises important clinical questions regarding arrhythmia risk in this setting. Future studies are needed to better define the incidence of AF following esophageal stenting, identify high-risk features, and evaluate the potential role of cardiac monitoring or prophylactic strategies in select patients.

## Conclusions

This case highlights an uncommon but clinically significant association between esophageal mass or malignancy and the onset of AF, particularly following esophageal stent placement. While AF is a well-recognized postoperative complication after esophagectomy or radiotherapy, its occurrence as a direct result of mechanical compression from a tumor or stent remains underreported. Clinicians should be aware of this potential complication, especially in patients with predisposing cardiac substrates. Early recognition and management of AF in this context are crucial, given the challenges of anticoagulation in patients with active gastrointestinal malignancy and bleeding risk. Further studies are warranted to evaluate the incidence, pathophysiology, and long-term outcomes of AF in patients undergoing palliative or therapeutic esophageal stenting.
